# Acridine orange exhibits photodamage in human bladder cancer cells under blue light exposure

**DOI:** 10.1038/s41598-017-13904-0

**Published:** 2017-10-26

**Authors:** Yi-Chia Lin, Ji-Fan Lin, Te-Fu Tsai, Hung-En Chen, Kuang-Yu Chou, Shan-Che Yang, Ya-Ming Tang, Thomas I.-Sheng Hwang

**Affiliations:** 1 Department of Urology, Shin Kong Wu Ho-Su Memorial Hospital, Taipei, Taiwan; 2 School of Medicine, Fu-Jen Catholic University, New Taipei City, Taiwan; 3 Central Laboratory, Shin Kong Wu Ho-Su Memorial Hospital, Taipei, Taiwan

## Abstract

Human bladder cancer (BC) cells exhibit a high basal level of autophagic activity with accumulation of acridine-orange(AO)-stained acidic vesicular organelles. The rapid AO relocalization was observed in treated BC cells under blue-light emission. To investigate the cytotoxic effects of AO on human BC cell lines under blue-light exposure, human immortalized uroepithelial (SV-Huc-1) and BC cell lines (5637 and T24) were treated with indicated concentrations of AO or blue-light exposure alone and in combination. The cell viability was then determined using WST-1, time-lapse imaging with a Cytosmart System and continuous quantification with a multi-mode image-based reader. Treatment of AO or blue-light exposure alone did not cause a significant loss of viability in BC cells. However, AO exhibited a dose-dependent increment of cytotoxicity toward BC cells under blue-light exposure. Furthermore, the tumor formation of BC cells with treatment was significantly reduced when evaluated in a mouse xenograft model. The photodamage caused by AO was nearly neglected in SV-Huc-1 cells, suggesting a differential effect of this treatment between cancer and normal cells. In summary, AO, as a photosensitizer, disrupts acidic organelles and induces cancer cell death in BC cells under blue-light irradiation. Our findings may serve as a novel therapeutic strategy against human BC.

## Introduction

Bladder cancer (BC) remains a commonly diagnosed urological malignancy with a high recurrence rate. The standard treatment for managing BC is a complete transurethral resection of the bladder tumor (TURBT). Intravesical instillation with chemotherapeutic agents or bacillus Calmette-Guerin (BCG) for non-muscle invasive BC is generally used as an adjuvant therapy after TURBT^1^. Despite previous efforts, approximately 30% of patients will experience recurrence and 10% will eventually progress^2^. The possible mechanisms for recurrence are newly growing lesions, inadequate resection, missed lesions and replantation of the resected tumors^3^. Therefore, novel therapeutic options are warranted in BC treatment.

Macro-autophagy (autophagy) is a catabolic process that degrades unnecessary intracellular metabolites, damaged organelles and proteins during nutrient deprivation or metabolic stress. Autophagy begins with the formation of double-membrane vesicles, known as autophagosomes, which engulf cytoplasmic constituents. The autophagosomes then fuse with lysosomes, where the sequestered contents undergo degradation and recycling^4^. Acridine orange (AO) is a lysotropic dye that accumulates in acidic organelles in a pH-dependent manner and is commonly used to identify acidic vesicular organelles (AVOs)^5^. Under AO staining, the cytoplasm and nucleoli fluoresce green, whereas the acidic compartments, such as lysosomes or autophagolysosomes, fluoresce bright-red or orange-red with blue-light excitation^6^.

We failed to detect autophagy when using AO as a vital staining dye in human BC cells in a previous study^7^. The red dots representing AVOs were sometimes missing and the intensity of red fluorescence was not increased in AO-stained BC cells, despite the confirmation of the existence of autophagy^7^. In addition, decreased cell viability was observed in AO-stained BC cells. This observation suggested that AO may exhibit cytotoxicity toward human bladder cancer cells even when treated with the regular dose that is commonly used to detect autophagy progression. AO, as a photosensitizer, has been shown to cause cell death of human fibroblasts upon excitation with blue-light^8^. It is possible that cellular damage occurred in AO-stained BC cells during the detection processes with blue-light exposure. In this study, we aimed to present the AO-mediated photodamage on human BC cells compared with human immortalized uroepithelial cells (SV-Huc1).

## Results

### AO vital staining did not reflect autophagy induction in human BC cells

**Figure 1:**
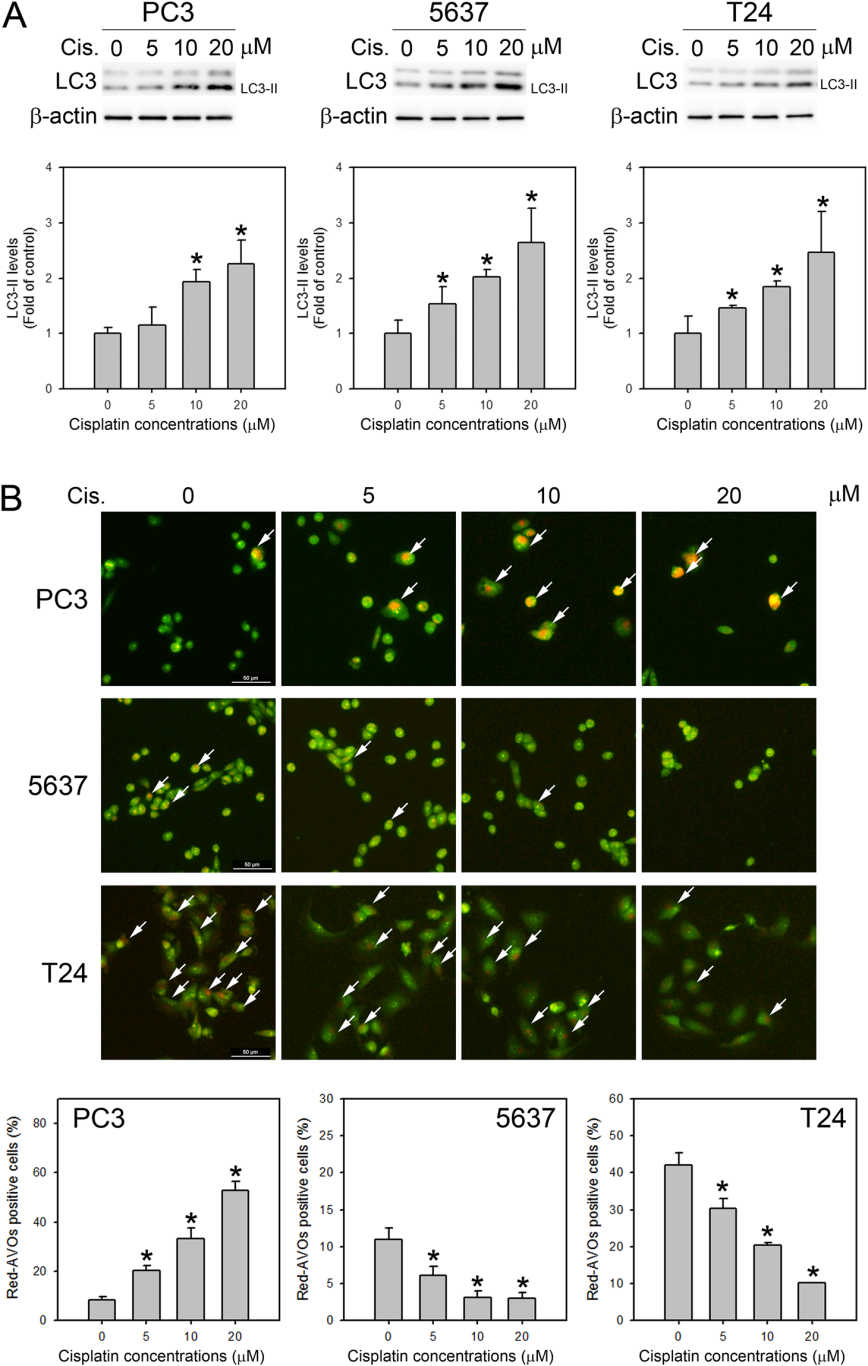
AO staining failed to detect cisplatin induced autophagy in bladder cancer cells. (**A**) The LC3-II processing in PC3, 5637 and T24 cells treated with indicated concentrations of cisplatin for 24 hrs was detected by Western blotting. The relative expression levels of LC3-II were quantified by densitometric scanning, and the results are presented in the lower histogram showing the fold-change relative to the DMSO-treated controls. (**B**) AO stained red fluorescent-positive AVOs were increased in cisplatin-treated PC3 cells but were decreased in cisplatin-treated 5637 and T24 cells. Red fluorescence (white arrows) indicated the AO-stained AVOs within the cells. All the images were taken under the same magnification, the scale bars (50 μm) were present in the first image in each raw. The percentage of red-AVO positive cells was calculated in 20 photos of each condition and the results are presented in the lower histogram. Representative blots and photos from three independent experiments with similar results are shown. *P < 0.05.

**Figure 2:**
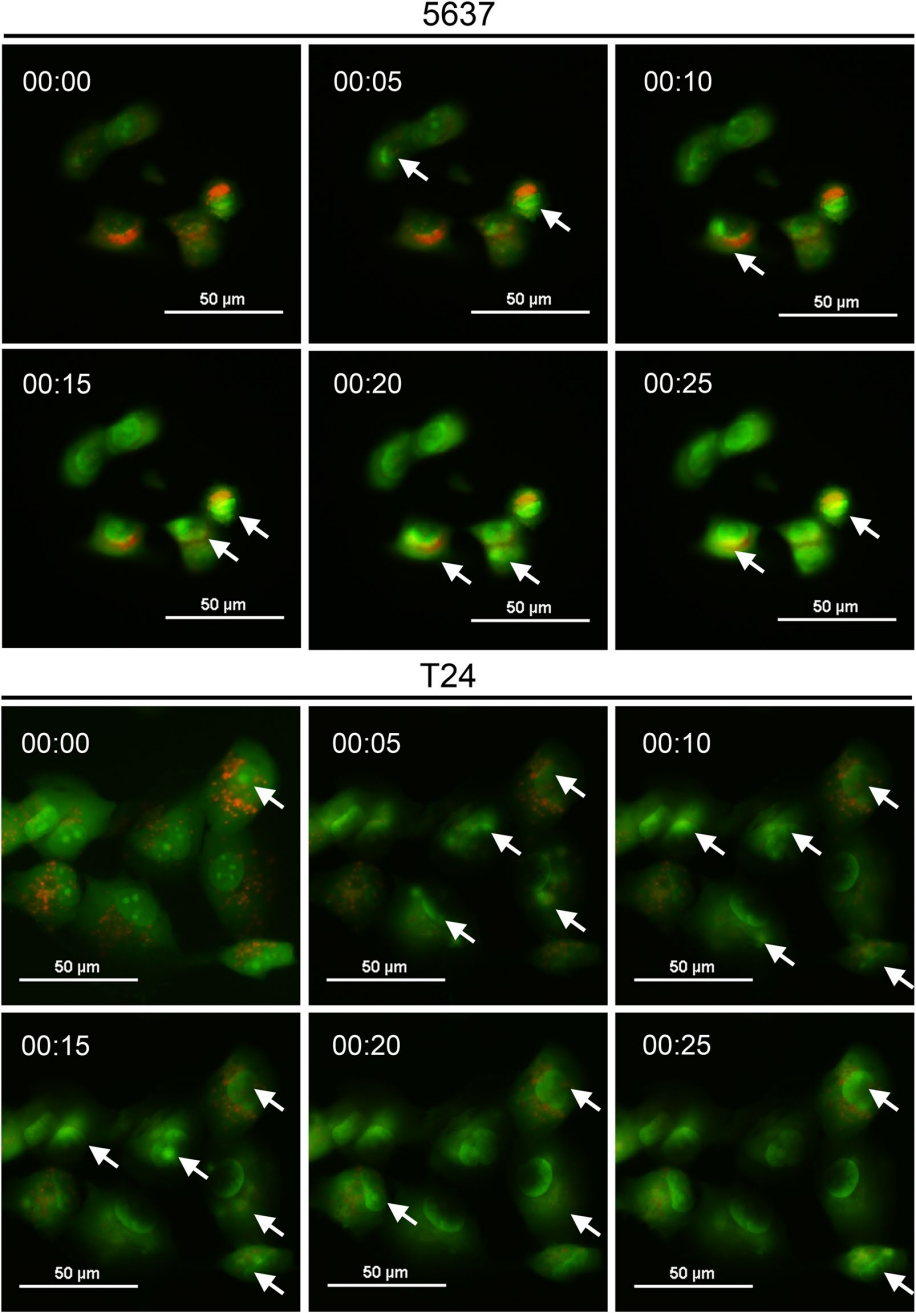
Rapid AO translocation under fluorescence microscopy. The (**A**) T24 and (**B**) 5637 cells seeded in 3 cm glass-bottom plates were treated with 1 μg/ml AO for 30 minutes and then images were recorded under fluorescence microscopy. Time-lapse images with 5-second intervals are shown. The video clips are given in supplementary video 1. Representative results from three independent experiments with similar results are shown. White arrows indicate AO translocation where red fluorescence decayed and green fluorescence increased. (Scale bars, 50 μm).

To demonstrate that AO vital staining could not reflect the autophagic status in human bladder cancer cells, we detected autophagy induction by cisplatin in prostate and bladder cancer cells. The PC3, 5637 and T24 cells were treated with 5, 10, and 20 μM cisplatin for 24-hr, and then the processing of an autophagic marker protein, LC3-II, was detected by Western blotting. As shown in Fig. 1A, the processing of LC3-II was detected in all three tested cell lines, suggesting that cisplatin treatment induces autophagy in these cells. However, when the cisplatin treated cells were incubated in the AO staining medium for 30 minutes and the medium was refreshed prior to imaging under fluorescence as described previously^6^, the percentage of red fluorescent-positive cells (which represent stained acidic vesicular organelles, AVOs) were increased only in PC3 cells (Fig. 2B). In 5637 and T24 cells with a high basal level of autophagic activities, the red fluorescent-positive cells were detected in controls, but the number of positive cells gradually decreased upon increasing the cisplatin concentrations. These results suggested that AO vital staining as an indication for detecting autophagy induction is not adequate in BC cells.

### Rapid AO translocation in T24 cells

To investigate why the percentage of AVO-positive cells was decreased in AO-stained BC cells, we observed the AO-stained BC cells under fluorescence microscopy. As shown in Fig. 2 (also see supplementary video 1), the 5-second time-lapse images clearly demonstrated that red fluorescence (which represents stained acidic vesicular organelles, AVOs) was diminished under blue-light transmitted from fluorescence microscopy, and green fluorescence increased within the cell. This phenomenon is referred to as AO translocation under blue-light exposure, according to a previous publication^8^.

### Treatment with AO or exposure to blue light alone did not exhibit cytotoxicity toward human BC cells

**Figure 3:**
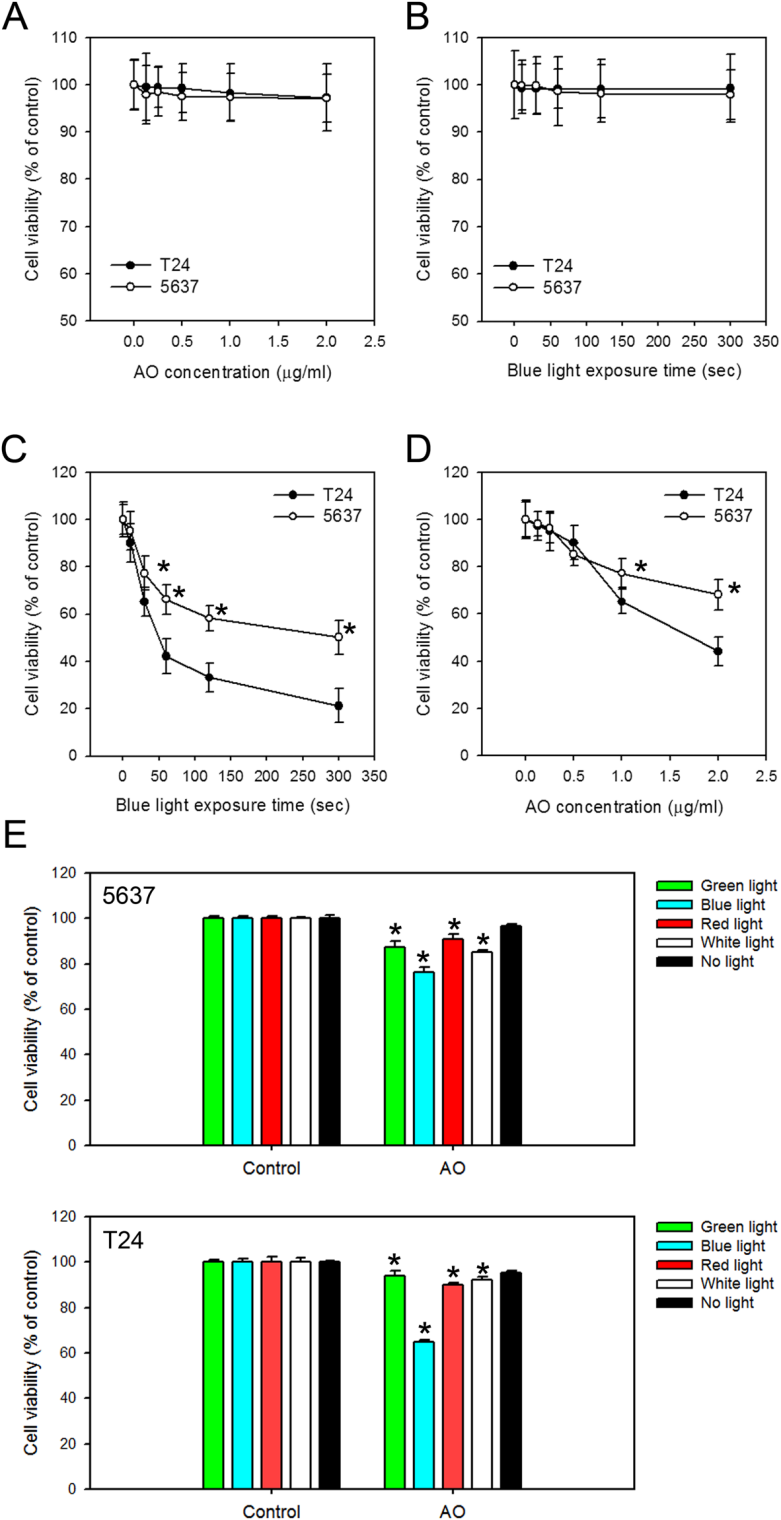
The effect of AO or light exposure on cell viability of human BC cells. Cell viability was assayed with 1 hr WST-1 incubation after 5637 or T24 cells were (**A**) treated with 0–2.5 μg/ml AO for 30 mins, (**B**) exposed to blue light for 0–300 seconds, (**C**) treated with 1 μg/ml AO for 30 mins and exposed to 0–300 seconds of blue light, (**D**) treated with 0–2 μg/ml AO for 30 mins and exposed to 30 seconds of blue light, or (**E**) treated with 1 μg/ml AO for 30 mins and exposed to 30 seconds of different light sources prior to the detection of cell viability. The values are shown as the mean ± S.D. of 3 independent experiments; *P < 0.05.

Decreased viability was observed in our previous attempts using AO staining for AVOs during autophagy detection^9^. We therefore were interested in whether AO or light exposure exhibited cytotoxicity in human BC cells. To address these questions, we developed light source devices with LED bulbs that emit blue, green, red and white lights, mimicking the cells under fluorescence microscopy (as described in Materials and Methods). We then investigated the effect of AO with or without light exposure on cell viability using 5637 and T24 cells. During the entire experimental process, including the addition of AO and WST-1, the samples were protected from environmental lights. As shown in Fig. 3A, no cell viability loss was detected in 5637 and T24 cells treated with different concentrations of AO for 30 mins, nor was cell viability loss detected in the controls. We next tested whether blue-light exposure alone has any effect on cell viability of BC cells. Cells were exposed to blue-light with different time intervals prior to the detection of cell viability, and the results showed that neither cancer cells nor control cells showed viability loss (Fig. 3B).

### Decreased cell viability and tumor formation in AO-stained BC cells exposed to blue-light *in vitro* and *in vivo*

Since AO staining or blue-light exposure alone had no impact on cell viability, we next investigated the effects of blue-light on AO-stained cells. Cells were first treated with 1 μg/ml AO (the dose that is commonly used to detect AVO formation when autophagy is induced) and were then exposed to blue-light with different intervals ranging from 0–5 mins. The results showed decreased cell viability in AO-stained cells starting at 30 seconds of exposure (Fig. 3C). To test whether the AO concentration had any effect on cell viability upon blue-light exposure, cells were treated with different concentrations of AO and exposed to blue-light for 30 seconds. As shown in Fig. 3D, decreased cell viability was observed when increasing the concentrations of AO with 30 seconds of blue light exposure. Based on these findings, we chose 1 μg/ml AO staining and 30 seconds of light exposure (referred as AO-photodynamic treatment, AO-PDT) for the following experiments. AO was originally used as a nucleic acid fluorescent cationic dye for its cell-permeable characteristic and ability to interact with DNA and RNA. When AO binds to DNA, it has a similar spectrum as fluorescein, with an excitation maximum at 502 nm and an emission maximum at 525 nm (green). The excitation maximum shifts to 460 nm and the emission maximum shifts to 650 nm (red) when AO is associated with RNA. AO is also used to stain acidic organelles non-specifically with a maxima excitation at 502 nm and an emission at 525 nm^10,11^. To test if other light sources other than blue light could induce AO translocation and decrease cell viability, we made a light box with green, red and white light LEDs. The 5637 and T24 cells were seeded in 96-well plates overnight prior to staining with 1 μg/ml AO for 30 mins. Then, the medium was refreshed and the plate was placed under different light sources for 30 secs. The cell viability was detected immediately after treatment by incubation with WST-1 reagent for an additional hour. As shown in Fig. 3E, although all light sources seemed to affect the cell viability in AO-stained cells, the lowest cell viability was observed in those exposed to blue light. Therefore, 1 μg/ml AO staining for 30 mins and blue light exposure for 30 secs was selected for the subsequent experiments.

**Figure 4:**
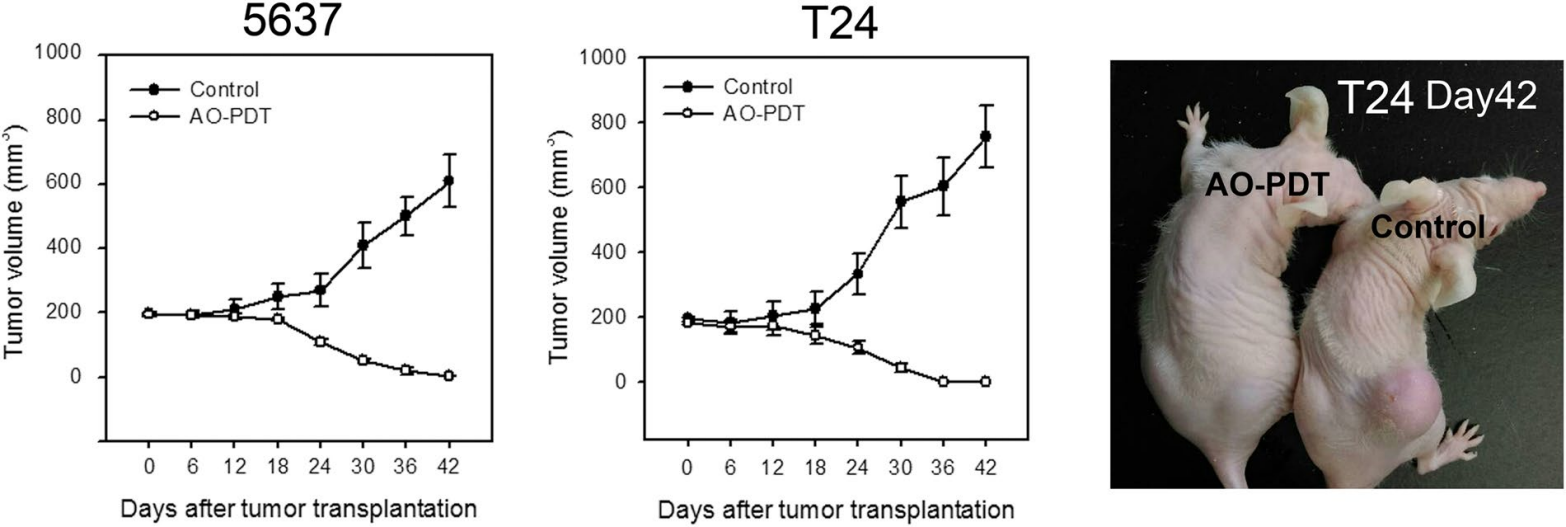
AO-PDT suppressed tumor formation in the xenograft mouse. The evaluation of (**A**) 5637 and (**B**) T24 tumor formation was performed in the nude mice. 5637 and T24 cells were treated with (AO-PDT group, n = 10 for each cells) or without (control group, n = 10 for each cells) AO-PDT prior to inoculation to the lower back of these mice. The tumor volume was detected in each mouse every 6 days beginning from the inoculation. The values are shown as the mean ± S.D. (**C**) A representative photo of tumor formation in a mouse bearing T24 cells with or without AO-PDT treatment is shown.

To test if tumor growth of 5637 and T24 was really compromised with AO-PDT treatment *in vivo*, 5637 and T24 xenograft tumor mouse models were used in the *in vivo* study. The 5637 and T24 cells with (AO-PDT group) or without (control group) AO-PDT treatment were transplanted to nude mice, and the tumor volume was recorded every 6 days. As shown in Fig. 4, AO-PDT significantly inhibited tumor growth. At the end of the experiment, 10 of 10 mice receiving AO-PDT treatment had no visible tumor mass at the injection side, while 9 of 10 mice and 8 of 10 mice in the control groups for 5637 and T24, respectively, had an enlarged tumor. These results suggested that AO-PDT treatment exhibited cytotoxicity toward human bladder cancer cells and inhibited tumor growth.

### AO-PDT treatment has minimal effects on cell viability in immortalized uroepithelial cells

**Figure 5:**
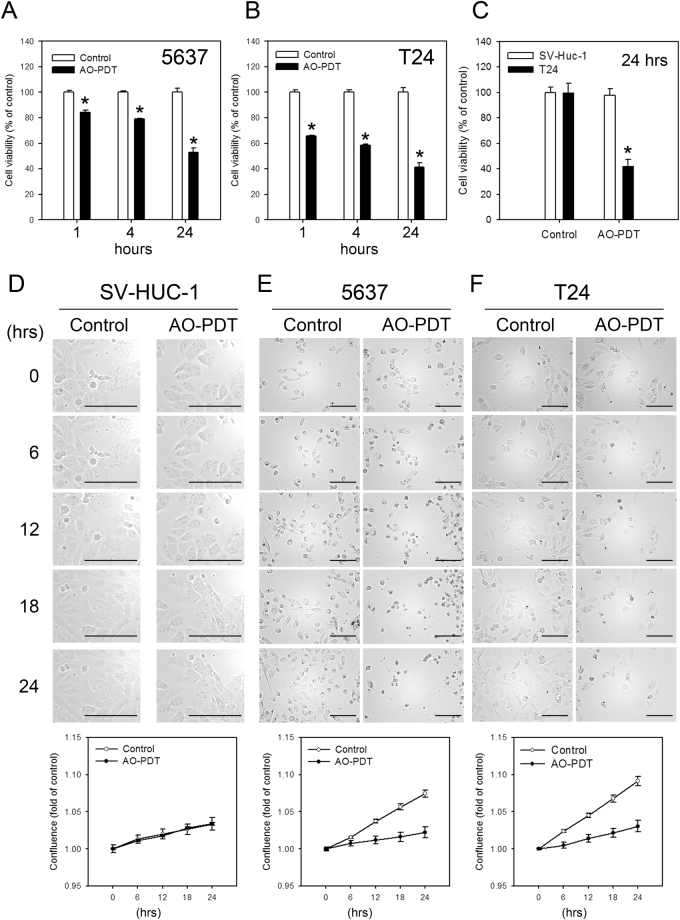
The AO-PDT treatment had a minimal impact on cell viability of SV-Huc-1 compared to T24 and 5637 cells. Cell viability was detected using WST-1 reagent in (**A**) T24 and (**B**) 5637 cells treated with AO-PDT (incubated with 1 μg/ml AO for 30 min, exposed to blue-light for 30 sec) and incubated further for 1, 4 and 24-hr. (**C**) Cell viability of SV-Huc-1 and T24 cells at 24-hr after AO-PDT treatment. Data are presented as the mean ± S.D. of 3 independent experiments. *P < 0.05. Time-lapse cell morphological images acquired automatically with a Cytation 5 Imaging Reader. Representative photos of (**D**) SV-Huc-1, (**E**) 5637, and (**F**) T24 cells treated with AO-PDT for 0, 6, 12, 18 and 24 hrs are shown. (Scale bars, 50 μm) Quantitative assessments of cell confluence in each condition were generated by the built-in software (Biotek), and the values corresponding to each data point are present in the bottom histogram. Representative results from three independent experiments with similar results are shown.

To understand if the photodamage caused by AO-PDT treatment is unique in cancer cells, the same treatment was performed in SV-Huc-1 cells. As shown in Fig. 5A,B, the decreased cell viability of the 5637 and T24 cells was observed at 1, 4 and 24-hr after AO-PDT treatment, whereas SV-Huc-1 exhibited no significant loss of viability at 24-hr after AO-PDT treatment, compared to the T24 cells (Fig. 5C). The time-lapse monitoring of cell morphology and viability was consistent with the findings that AO-PDT did not reduce cell viability in SV-Huc-1 cells (Figs 5D and S1; and supplementary videos 2–4). These data suggested that AO-PDT treatment is effective in BC cells but has minimal impact on normal cells.

### AO-PDT treatment induces necrotic and apoptotic cell death in uroepithelial cells

**Figure 6:**
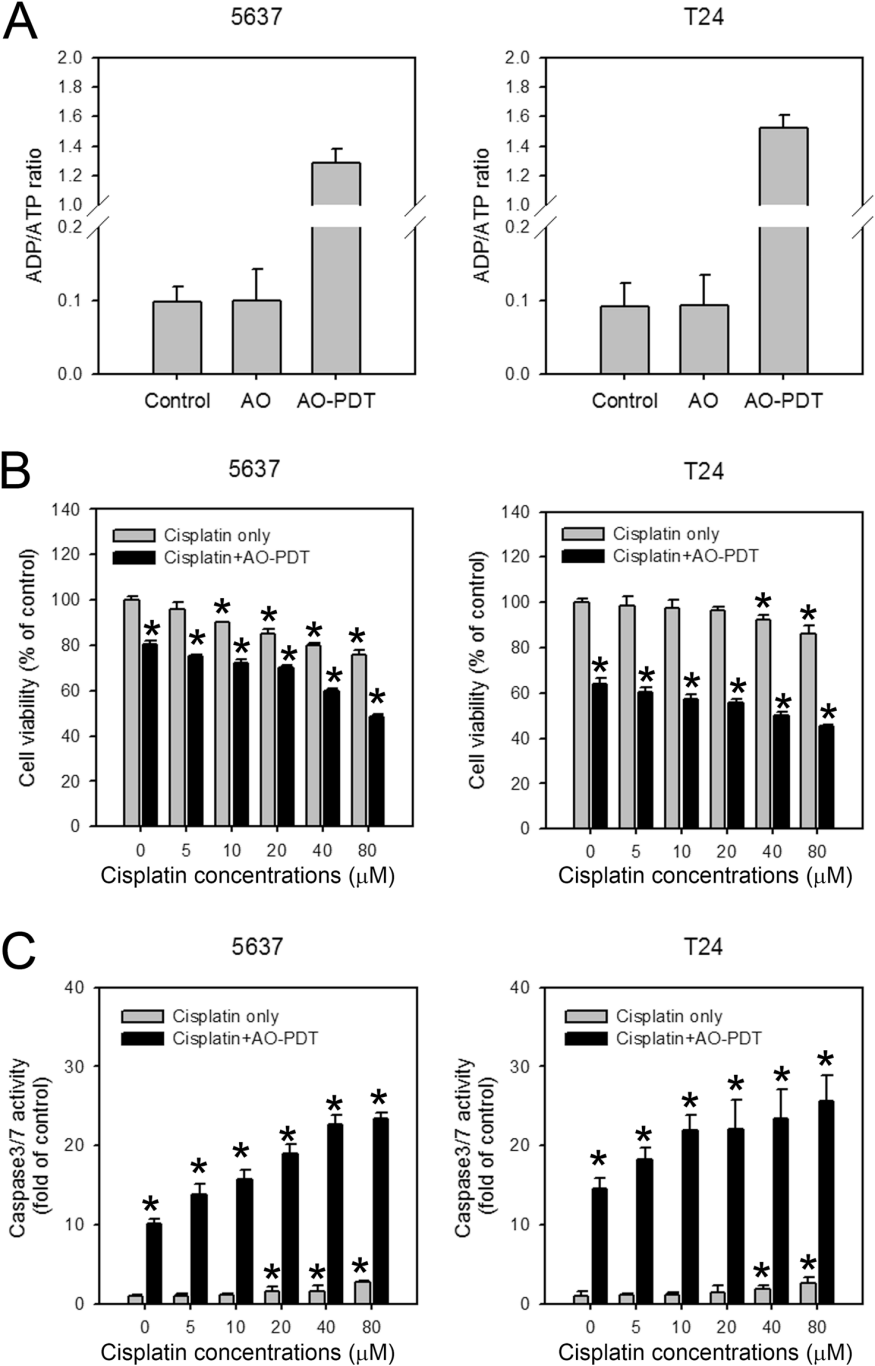
The AO-PDT treatment induced necrotic and apoptotic cell death in 5637 and T24 cells. (**A**) Increased ADP/ATP ratio in AO-PDT treated cells. The 5637 and T24 cells were treated with AO alone for 30 mins, or with AO-PDT. (**B**) The cell viability and (**C**) caspase 3/7 activities were detected in cells treated with the indicated concentrations of cisplatin for 24 hrs with or without AO-PDT treatment. The results are the mean ± S.D. of 3 independent experiments performed in 8 duplicated wells. *P < 0.05.

The cell viability decreased rapidly in cells treated with AO-PDT. It is likely that AO-PDT induces necrotic cell death because the loss of viability was detected only one hour after treatment. As shown in Fig. 5A,B, approximately 20 and 40% cell loss was observed in 5637 and T24 cells, respectively, treated with AO-PDT. Therefore, we detected the ADP/ATP ratio in these cells. In the healthy viable cells, the ADP/ATP ratio was expected to be below 0.1. When the ADP/ATP ratio increased to 1.0, the cells underwent apoptosis. Due to the dissipation of intracellular energy levels that occurs in necrosis, the ADP/ATP ratio reached higher values^12^. As shown in Fig. 6A, a significant amount of ADP accumulation was observed in the AO-PDT treated cells (Fig. 6A), suggesting necrotic cell death in at least a portion of the treated cells. However, cell viability decreased overtime (Fig. 5) even when the medium was refreshed. Therefore, apoptotic cell death may also be responsible for the loss of cell viability in AO-PDT treated cells. To test this theory, cell viability and the activation of caspase 3/7 activity in 5637 and T24 cells treated with various concentrations of cisplatin for 24-hr with or without AO-PDT treatment were detected. As demonstrated in Fig. 6B,C, the caspase 3/7 activities in control cells (without cisplatin treatment) were increased, suggesting AO-PDT induced apoptosis. The results also suggested that AO-PDT treatment could be combined with current anticancer drugs to enhance the cytotoxicity effect against BC.

## Discussion

In addition to maintaining cellular homeostasis, autophagy has been shown to play an important role in tumor formation and progression^13,14^. Treatment resistance and tumor dormancy were demonstrated in tumor cells resulting from stress-induced autophagy that leads to tumor regrowth and progression^15^. Accumulating studies in preclinical models showed that inhibition of pro-survival autophagy kills tumor cells by enhancing apoptotic cell death^4,16^. We previously reported that human BC cell lines exhibit a high basal level of autophagic activity^9^. Inhibition of basal autophagic activity decreased cancer cell viability by enhancing apoptosis, and our finding is consistent with the studies conducted in pancreatic and lung cancer cells^17,18^.

The AO, as a weak basic and cell-permanent dye, was first extracted from coal tar over a hundred years ago. It has a low molecular weight and therefore can rapidly diffuse into the cytoplasm of living cells within a few seconds to bind to the DNA, RNA and acidic vesicles such as lysosomes and others in the cells. It emits red fluorescence when bound to single-strand DNA or RNA and green fluorescence when bound to double-strand DNA^19^. AO was also demonstrated to be a pH indicator, photosensitizer, antitumor drug, and a detector of apoptosis, sperm mobility, bacteria and parasites^20–24^. The anticancer activity of AO was first demonstrated in a rodent model in the early 1950s^20,25^. It has been reported to induce apoptosis in cultured human fibroblasts by photo-oxidative disruption of lysosomal membranes when cells were stained with 5 μg/ml AO followed by blue-light irradiation^8^. In addition, a series of reports from Uchida A. and colleagues demonstrated the successful use of AO-PDT or AO-RDT (radiodynamic therapy) in human musculoskeletal sarcomas^26–30^. They also determined the LD50 of AO in mice intravenously and concluded that AO is safe for use as cancer therapy when it is kept at 1.0 mg/kg or below^31^.

In this study, we reported for the first time the effect of AO-PDT in cultured human BC cell lines and its inhibition on tumor growth of the high-grade 5637 and T24 cells in a xenograft mouse model. First, we demonstrated that AO is not a good indicator for cisplatin-induced autophagy in BC cells (Fig. 1). Next, we discovered that AO translocation rapidly occurred in AO-stained BC cells under blue-light exposure (Fig. 2). It is possible that photooxidative disruption of lysosomal membranes leads to the induction of apoptotic cell death in these AO-PDT treated BC cells, as reported previously^8^. We found the decreased cell viability of BC cells was dependent on the concentration of AO and the exposure time of blue-light (Fig. 3). The AO-PDT-treated 5637 and T24 cells were unable to grow in the xenograft model (Fig. 4). The cytotoxicity caused by AO-PDT was significantly profound in 5637 and T24 cells compared to SV-Huc-1 (Figs 5 and S1). Furthermore, the increased ratio of ADP/ATP and the enhanced caspase 3/7 activity in AO-PDT treated cells suggested both necrotic and apoptotic cell death in these cells (Fig. 6). In addition, our previous study demonstrated that cisplatin induces protective autophagy as a resistant mechanism in BC cells^32^. In the current study, we found AO-PDT treatment significantly enhanced cytotoxicity in 24-hr cisplatin-treated BC cells (Fig. 6B,C), indicating the potential for combining AO-PDT with conventional chemotherapeutic agents. As AO is easily attracted to low-pH organelles^33^, it is possible that BC cells exhibit elevated levels of autophagy providing much more acidic vesicular organelles (AVOs) as targets for AO accumulation and therefore AO-PDT is more effective in cancer compared to normal cells. Our findings were consistent with the report that AO selectively accumulated in tumor tissues in human musculoskeletal sarcomas compared to the adjacent normal tissue, because the tumor cells were more acidic due to the accelerated metabolic rates^26,34^.

Unlike the case for human musculoskeletal sarcomas, AO-PDT treatment for human BC could be easily applied using intravesical infusion with AO and introduction of the blue-light by currently existing cystoscopy (for photodynamic diagnosis or narrow-band image). Therefore, future development of AO-PDT in clinical therapy against human BC is highly possible. In addition, the International Agency for Research on Cancer (IARC) of the WHO reported that AO was considered to be non-carcinogenic for humans^35^. Therefore, intravesically introducing AO-PDT treatment not only avoided systematic damage, as other chemotherapeutic agents do, but provided targeted effects on cancer cells without harming adjacent normal cells when applied for a short period of time. We plan to investigate the mechanisms of cell death (apoptosis or necrosis) involved in AO-PDT treatment using human BC cells and a bladder cancer orthotopic model for future development of novel therapeutic strategies using PDT against human BC.

There are some limitations in this study. First, it was a cell-based study. Although we demonstrated the inhibition of tumor growth in the 5637 and T24 xenograft models, using an orthotopic model bearing bladder tumors to test the effect of AO-PDT on the inhibition of tumor growth may represent a more suitable preclinical model. The wavelength spectrum and the energy and strength of blue-light requires further studies for the development of specific and effective light sources for clinical applications. In addition, the experiments regarding lysosomal or mitochondrial damage caused by AO-PDT that induces necrotic or apoptotic cell death requires further investigation.

AO-PDT was demonstrated to effectively decrease the viability of human BC cells *in vitro* and *in vivo* for the first time, with a minimal effect on immortalized uroepithelial cells. Our results provided basic knowledge and strategies for future clinical applications using AO-PDT against human BC.

## Methods

### Chemicals, Reagents and Cell cultures

All the chemicals were purchased from Sigma-Aldrich (St Louis, MO, USA) and prepared with standard procedures as a stock solution. The human immortalized uroepithelial cell line (SV-Huc1), bladder cancer cell lines (5637 and T24), and PC3 prostate cancer cells were obtained from the Bioresource Collection and Research Center (BCRC; Hsinchu, Taiwan). Cells were seeded on plastic plates (96-well or 6-well) or 3-cm glass bottom dishes for cell viability assays or fluorescent imaging, respectively.

### Detection of autophagy by monitoring LC3-II processing

The PC3, 5637 and T24 cells were treated with 0–20 μM cisplatin for 24 hrs and subjected to protein extraction. The processing of LC3-II was detected by Western blotting as previously described^32^.

### Fluorescent imaging of AO translocation

The PC3, 5637 and T24 cells with or without the 0–20 μM cisplatin treatment were seeded in 3-cm glass bottom dishes for 24 hrs and were exposed to AO staining medium (1 μg/ml in complete medium) for 30 min. The AO-staining medium was refreshed prior to the subsequent experiments. All the treatment processes were performed with avoidance of environmental light. The dishes were immediately wrapped with aluminum foil and subjected to fluorescent imaging as described previously^6^. The Nikon inverted microscope Eclipse Ti-E (Nikon, Kobe, Japan) equipped with a 130-W fluorescence light source was used. The filter block was used that contained a 465- to 495-nm bandpass excitation filter, 505-nm dichroic mirror and 590-nm long-pass barrier filter. The percentage of cells with red-fluorescence (indicating AVOs) was calculated using 20 photos in each condition. Time-lapse sequences were recorded with a Nikon color CMOS Camera -DS-Ri2, and the time duration for each time-lapse sequence was 30 seconds. A single image from the indicated time point was isolated using Nikon NIS-Elements Advanced Research software. Six time-lapse images with 5-second intervals were extracted from the video.

### Home-made device for blue/red/green/white-light sources

To build a blue-light source, the blue-light LED bulbs (with a peak wavelength of 443.7 and energy of 55.28 lumen per LED in a 6-cm distance) were chosen for their production of less heat compared to the conventional mercury lamp. Six LED bulbs were welded to a small electronic board and attached to a cover made by a cardboard box (Fig. S2A) with the dimensions of 15 cm (length) × 12 cm (width) × 6 cm (height). The LEDs were driven by a commercially available power converter (Fig. S2B). The space under the cover was accessible for one 96-well plate or 10-cm culture dish (Fig. S2C). With the same design, we also build light boxes with green (peak wavelength, 565 nm), red (peak wavelength, 633 nm) and white (multichip white) LED bulbs to create different light sources.

### Cell viability assays

Cells were seeded in 96-well plates prior to the indicated treatment. Cell viability was assayed using WST-1 reagent (Roche Diagnostics, Mannheim, Germany) as described previously^36^ and was determined as the percentage of the controls. Samples were protected from light during the assays and the incubation time for WST-1 was set to 1 hr. Each condition was performed in eight replicate wells, and the data represented at least three independent experiments.

### Time-lapse monitoring of cell morphology and viability

The SV-Huc-1, 5637, and T24 cells seeded in octuplicates in 96-well plates were treated with or without 1 μg/ml AO for 30 min, and exposed to blue light for 30 sec (AO-photodynamic treatment, AO-PDT). The AO-staining medium was refreshed with complete medium, and the plates were incubated within Cytation 5 (BioTek Instruments, Inc., Winooski, VT, USA). The data acquisition was performed each hour for 24-hr. Live cell imaging (CytoSmart System, Lonza, Visp, Switzerland) was used to record real-time cell morphology in control or AO-PDT-treated cells. Control and treated-SV-Huc-1, −5637 or -T24 cells were constantly and automatically monitored in 15 min intervals for 36 hrs. The time-lapse photos as well as cell viability data were generated by the built-in software of each instrument.

### Animal experiments

Forty athymic BALB/c (nu/nu) mice (5 weeks old) were purchased from BioLASCO Taiwan Co., Ltd. (http://www.biolasco.com.tw). Overnight cultured 5637 and T24 cells at 70–80% confluency were treated with (test group) or without (control group) AO-PDT. Cells were then trypsinized, collected and counted. Collected cells (5.0 × 10^6^) suspended in 100 μl of RPMI 1640 medium mixed with 100 μl Matrigel (Becton Dickinson, NJ, USA) were transplanted subcutaneously into the backs of these mice, with 10 mice in each group. The tumor size was measured every 6 days beginning right after tumor inoculation and was calculated using the following formula: volume (a rotational ellipsoid) = L × S^2^ × 0.5236, where L is the long axis and S is the short axis. All animal experiments were performed according to a protocol approved by the Institutional Animal Care and Use Committees (IACUC) of Shin-Kong Wu Ho-Su Memorial Hospital (Taipei, Taiwan).

### Measurement of the ADP/ATP ratio

The ADP/ATP ratio was determined using a commercially available kit (Sigma-Aldrich). The assay was based on measurement of the adenylate nucleotides and allowed the assessment of the mode of cell death^12^. In brief, the 5637 and T24 cells seeded in octuplicates in white clear-bottom 96-well plates were treated with or without 1 μg/ml AO for 30 min and exposed to blue light for 30 sec (AO-photodynamic treatment, AO-PDT). The AO-staining medium was refreshed with complete medium, and the plates were incubated for another hour. After treatment, cells were lysed in ATP assay buffer and processed for ATP and ADP determination following the kit’s instructions. The ADP/ATP ratio was calculated using a formula provided by the manufacturer. Luminescence was measured using a microplate reader (Victor X2, Perkin Elmer Life Sciences, Waltham, MA, USA).

### Caspase 3/7 activity assay

The activation of caspase-3/7 was determined using the substrate (Z-DEVD)_2_-R110 (Bachem, Torrance, CA, USA) as described previously^37^. Briefly, cell viability in cells treated with indicated concentrations of cisplatin for 24 hrs with or without AO-PDT treatment was measured using WST-1 reagent as described above. Subsequently, the caspase 3/7 activity was detected in the cells by adding assay buffer containing (Z-DEVD)_2_-R110 substrate and incubated at 37 °C for 1 hr. The fluorescence intensity of the proteolytically released fluorophore R110 was then measured using a plate reader (Victor X2) at an excitation wavelength of 485 nm and emission wavelength of 535 nm.

### Statistics

All data were collected from experiments that were performed at least three times and were expressed as the mean ± standard deviation (SD). Student’s t-test was performed to calculate the statistical significance of the differences in variables between groups and p < 0.05 was considered statistically significant.

## Electronic supplementary material

supplementary data

supplementary video 1

supplementary video 2

supplementary video 3

supplementary video 4
