# Supplementary material for: Acridine orange exhibits photodamage in human bladder cancer cells under blue light exposure
Source: Sci Rep. 2017 Oct 26;7:14103. doi: 10.1038/s41598-017-13904-0 (PMC5658329; doi:10.1038/s41598-017-13904-0)
Supplement: Supplementary file 1 — supplementary data [file 41598_2017_13904_MOESM1_ESM.pdf]

Article title: Acridine orange exhibits photodamage in human bladder cancer cells under blue light exposure

Authors: Yi-Chia Lin, Ji-Fan Lin, Te-Fu Tsai, Hung-En Chen, and Kuang-Yu Chou, Shan-Che Yang, Ya-Ming Tang, and Thomas I-Sheng Hwang

Supplementary figures:

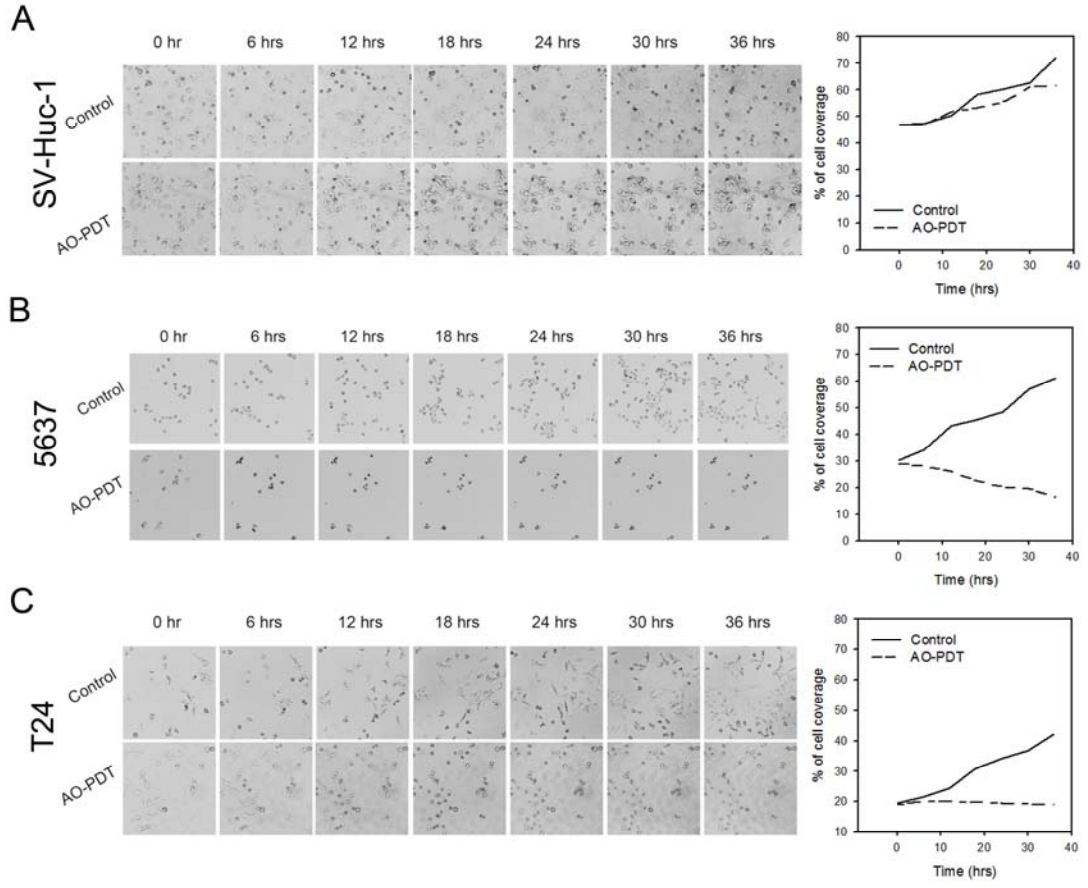

Fig. S1. Time-lapse cell morphological images recorded by the CytoSmart system. Cells were incubated with refreshed medium after AO-PDT treatment. Representative photos of (A) SV-Huc-1, (B) 5637 and (C) T24 cells treated with or without AO-PDT for 0, 6, 12, 18, 24, 30, and 36 hrs are shown. The composed videos for each cell are provided as supplementary video 2-4. Quantitative assessment of cell coverage in each condition was generated by an on-line analytic software (Lonza, Walkersville, MD, USA), and the values corresponding to each data point were present in the right histogram. Representative results from three independent experiments with similar results are shown.

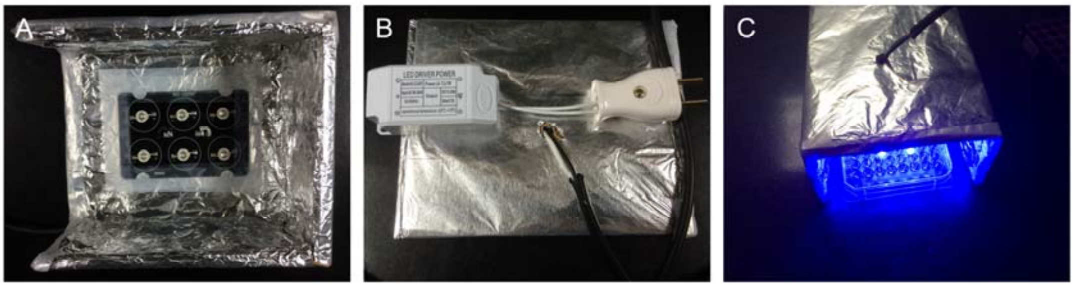

Fig. S2. Home-made blue-light box. (A) Six LED bulbs were welded to a small electronic board and attached to a cover box made of cardboard wrapped with aluminum foil with the dimensions of 15x12x6 cm (LxWxH). (B) The LED bulbs are driven with a commercially available power converter. (C) One 96-well plate fit in the box for blue-light exposure.

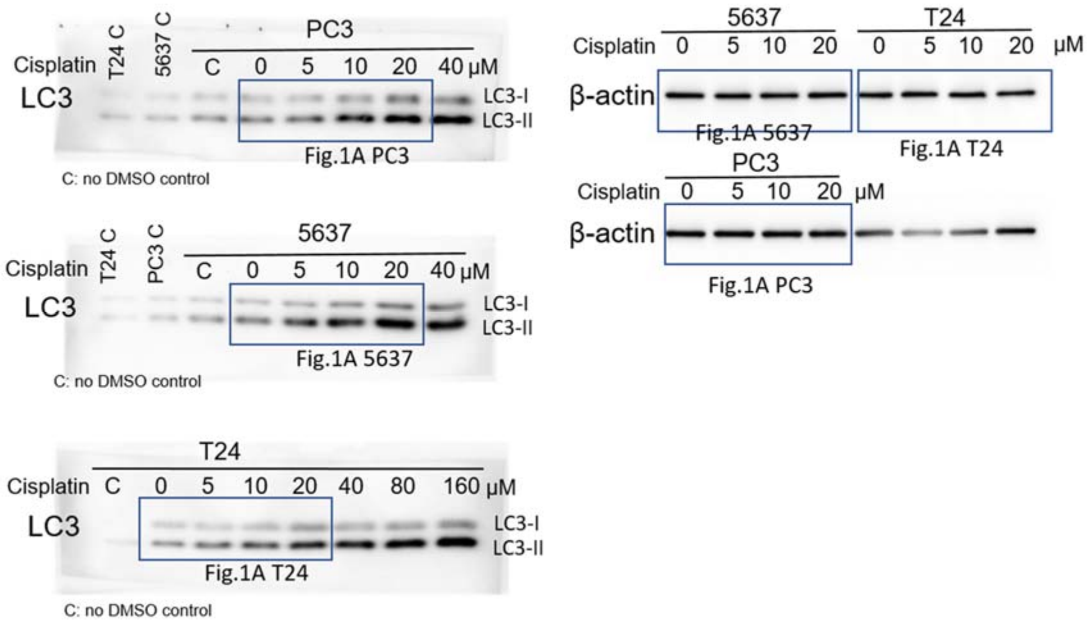

Fig. S3. Raw data of immunoblots in Fig 1. To detect LC3 expression, cells subjected to the indicated treatments were harvested and lysed, and the protein concentration was determined using a BCA protein assay (Pierce, Rockford, IL, USA). Proteins were separated via 14% sodium dodecyl sulfate polyacrylamide gel electrophoresis (SDS-PAGE) and were transferred to polyvinylidene difluoride (PVDF) membranes (Millipore, Billerica, MA, USA). Membranes were cut according to a protein marker (PageRuler Prestained Protein Ladder, Thermo Scientific) from 10-25 kDa and 35-55 kDa for detecting LC3 and β-actin, respectively. The membranes were than probed with antibodies against LC3 (LC3B (D11) XP® Rabbit mAb, #3868, Cell Signaling Technology) or β-actin (Monoclonal Anti-β-Actin antibody, #A5441, Sigma-Aldrich). Subsequent immunoblotting procedures were performed using a chemiluminescence process (Millipore) as per the manufacturer's instructions. The image acquisition and analysis were performed using a ChemiDoc-It camera system (ChemiDoc-ot 815, UVP, Upland, CA, USA). Cropped areas used in Fig.1A were boxed.

Supplementary video legends:

Supplementary video 1. Real-time monitoring of Rapid AO translocation under fluorescence microscopy. The 5637 (left) and T24 (right) cells were seeded in 3 cm glass-bottom plates and treated with 1 μg/ml AO for 30 minutes, the medium was refreshed, and then images were recorded under fluorescence microscopy as described in the Materials and Methods. The video clips of 5637 and T24 cells (each 29 seconds) were then composed into one video frame using image processing software, PowerDirector version 10.0 (Cyberlink Corp., New Taipei City, Taiwan). Representative results from three independent experiments with similar results are shown.

Supplementary video 2-4. Real-time imaging of cells with or without AO-PDT treatment for 36 hours. The growth of 5637 (videoS2), T24 (videoS3) and SV-Huc-1 (videoS4) cells with or without AO-PDT treatment was accessed in a real-time fashion for 36 hours using the CytoSmart System (Lonza). The videos were composed by extracted photos with the same sequence number of each experiment from the on-line analytic tools provided by Lonza. The videos from the control (in the left side of the video) and AO-PDT (in the right side of the video) groups in each cell were synchronized and composed to one video frame for comparison using image processing software, PowerDirector version 10.0 (Cyberlink Corp., New Taipei City, Taiwan). Representative results from three independent experiments with similar results are shown.
